# MRI-based habitat imaging predicts high-risk molecular subtypes and early risk assessment of lower-grade gliomas

**DOI:** 10.1186/s40644-025-00838-4

**Published:** 2025-03-28

**Authors:** Xiangli Yang, Wenju Niu, Kai Wu, Guoqiang Yang, Hui Zhang

**Affiliations:** 1 Department of Radiology, First Hospital of Shanxi Medical University, Taiyuan, 030001 China; 2 Shanxi Bethune Hospital, Third Hospital of Shanxi Medical University, Shanxi Academy of Medical Sciences, Tongji Shanxi Hospital, Taiyuan, 030032 China; 3 College of Medical Imaging, Shanxi Medical University, Taiyuan, 030001 China; 4 Department of Information Management, First Hospital of Shanxi Medical University, Taiyuan, 030001 China; 5 Shanxi Key Laboratory of Intelligent Imaging and Nanomedicine, First Hospital of Shanxi Medical University, Taiyuan, 030001 China; 6 Intelligent Imaging Big Data and Functional Nano-imaging Engineering Research Center of Shanxi Province, First Hospital of Shanxi Medical University, Taiyuan, 030001 China

**Keywords:** Glioma, MRI, Radiomics, Habitat, High-risk molecular subtypes, Prognosis

## Abstract

**Background:**

In lower-grade gliomas (LrGGs, histological grades 2–3), there exist a minority of high-risk molecular subtypes with malignant transformation potential, associated with unfavorable clinical outcomes and shorter survival prognosis. Identifying high-risk molecular subtypes early in LrGGs and conducting preoperative prognostic evaluations are crucial for precise clinical diagnosis and treatment.

**Materials and methods:**

We retrospectively collected data from 345 patients with LrGGs and comprehensively screened key high-risk molecular markers. Based on preoperative MRI sequences (CE-T1WI/T2-FLAIR), we employed seven classifiers to construct models based on habitat, radiomics, and combined. Eventually, we identified Extra Trees based on habitat features as the optimal predictive model for identifying high-risk molecular subtypes of LrGGs. Moreover, we developed a prognostic prediction model based on radiomics score (Radscore) to assess the survival outlook of patients with LrGGs. We utilized Kaplan-Meier (KM) survival analysis alongside the log-rank test to discern variations in survival probabilities among high-risk and low-risk cohorts. The concordance index was employed to gauge the efficacy of habitat, clinical, and amalgamated prognosis models. Calibration curves were utilized to appraise the congruence between the anticipated survival probability and the actual survival probability projected by the models.

**Results:**

The habitat model for predicting high-risk molecular subtypes of LrGGs, achieved AUCs of 0.802, 0.771, and 0.768 in the training set, internal test set, and external test set, respectively. Comparison among habitat, clinical, combined prognostic models revealed that the combined prognostic model exhibited the highest performance (C-index = 0.781 in the training set, C-index = 0.778 in the internal test set, C-index = 0.743 in the external test set), followed by the habitat prognostic model (C-index = 0.749 in the training set, C-index = 0.716 in the internal test set, C-index = 0.707 in the external test set), while the clinical prognostic model performed the worst (C-index = 0.717 in the training set, C-index = 0.687 in the internal test set, C-index = 0.649 in the external test set). Furthermore, the calibration curves of the combined model exhibited satisfactory alignment when forecasting the 1-year, 2-year, and 3-year survival probabilities of patients with LrGGs.

**Conclusion:**

The MRI-based habitat model simultaneously achieves the objectives of non-invasive prediction of high-risk molecular subtypes of LrGGs and assessment of survival prognosis. This has incremental value for early non-invasive warning of malignant transformation in LrGGs and risk-stratified management.

**Supplementary Information:**

The online version contains supplementary material available at 10.1186/s40644-025-00838-4.

## Introduction

Lower-grade gliomas (LrGGs) refer to brain gliomas pathologically graded as level 2–3 [1], constituting a highly complex tumor category. Historically, LrGGs were regarded as tumors with relatively favorable prognosis and mild biological behavior, leading to conservative clinical approaches [2]. However, long-term clinical follow-ups have revealed a minority subset within LrGGs with malignant transformation potential, exhibiting clinical courses akin to malignant glioblastomas and poor survival outcomes, irrespective of their pathological grading [3, 4]. This discovery shattered traditional perceptions of LrGGs, gradually recognizing the complexity and diversity of their biological behavior. In 2021, the World Health Organization’s classification guidelines for central nervous system tumors explicitly identified four high-risk molecular subtypes within LrGGs: IDH wild-type with TERT promoter mutation, IDH wild-type with EGFR amplification, IDH wild-type with + 7/-10 chromosomal alterations, and IDH mutation with CDKN2A/B homozygous deletion. Simultaneously, these four high-risk molecular subtypes were directly categorized as grade 4 gliomas, signaling their malignant clinical course and unfavorable survival prognosis [5, 6]. Therefore, non-invasive preoperative identification of high-risk molecular subtypes within LrGGs holds significant clinical significance for early detection of malignant transformation and timely intervention selection.

The molecular subtyping diagnosis of LrGGs often relies on invasive tissue biopsies and molecular testing, which increase the physical, psychological, and financial burden on patients [7, 8]. Previous studies have demonstrated the feasibility of using MRI-based radiomics to predict molecular subtypes of gliomas and have achieved promising results. Nevertheless, there are certain constraints to consider. Gliomas are known for their complex heterogeneity [9–12], while traditional radiomics typically treats gliomas as homogeneous entities. Quantitative feature extraction often occurs across the entire tumor region, which fails to capture the spatial heterogeneity of the tumor, leading to shortcomings in glioma assessment [13–15]. Recently, habitat technologies have made significant strides in overcoming these challenges. Specifically, the habitat imaging technology rooted in Darwinian dynamics can re-segment areas with distinct pathophysiological features based on the radiomic characteristics of lesions, resulting in the creation of multiple tumor subregions. This advancement aids in uncovering and investigating the spatial heterogeneity within tumors, offering a promising path for more precise, non-invasive prediction of molecular subtypes in LrGGs and enabling early risk stratification management.

Habitat analysis technology, rooted in Darwinian evolutionary dynamics, posits that each sub-habitat undergoes continuous evolution in both temporal and spatial dimensions [16–20]. Each tumor is not simply a homogeneous entity but exists as an ecosystem within multiple unique microenvironmental subregions. Habitat imaging technology visualizes the different habitat environments of tumors, establishing clear and predictable connections between macroscopic tumor features observed through imaging and the molecular, cellular, and microenvironmental characteristics of microscopic cancer cell populations [21]. It is particularly suitable for measuring the temporal and spatial heterogeneity within tumors [22–24], aiding in a deeper understanding of the evolutionary dynamics of glioma development and progression.

In this investigation, we formulated a preoperative MRI-derived habitat model aimed at concurrently predicting high-risk molecular subtypes and assessing survival prognosis for LrGGs. This is of significant importance for early non-invasive warning of malignant transformation in LrGGs, preoperative risk stratification management, and precision diagnosis and treatment.

## Materials and methods

### Patient data collection

This study adopted a multicenter research approach, retrospectively collecting data from three tertiary hospitals in our province (Hospital 1, Hospital 2, Hospital 3) from 2012 to 2023 and TCGA database. All three participating hospitals are affiliated with Shanxi Medical University. The research protocol obtained ethical approval from the Institutional Review Board of Shanxi Medical University (2021-K-K073). Given the retrospective nature of the study and the use of anonymized patient data, and the informed consent was waived.

**Fig. 1:**
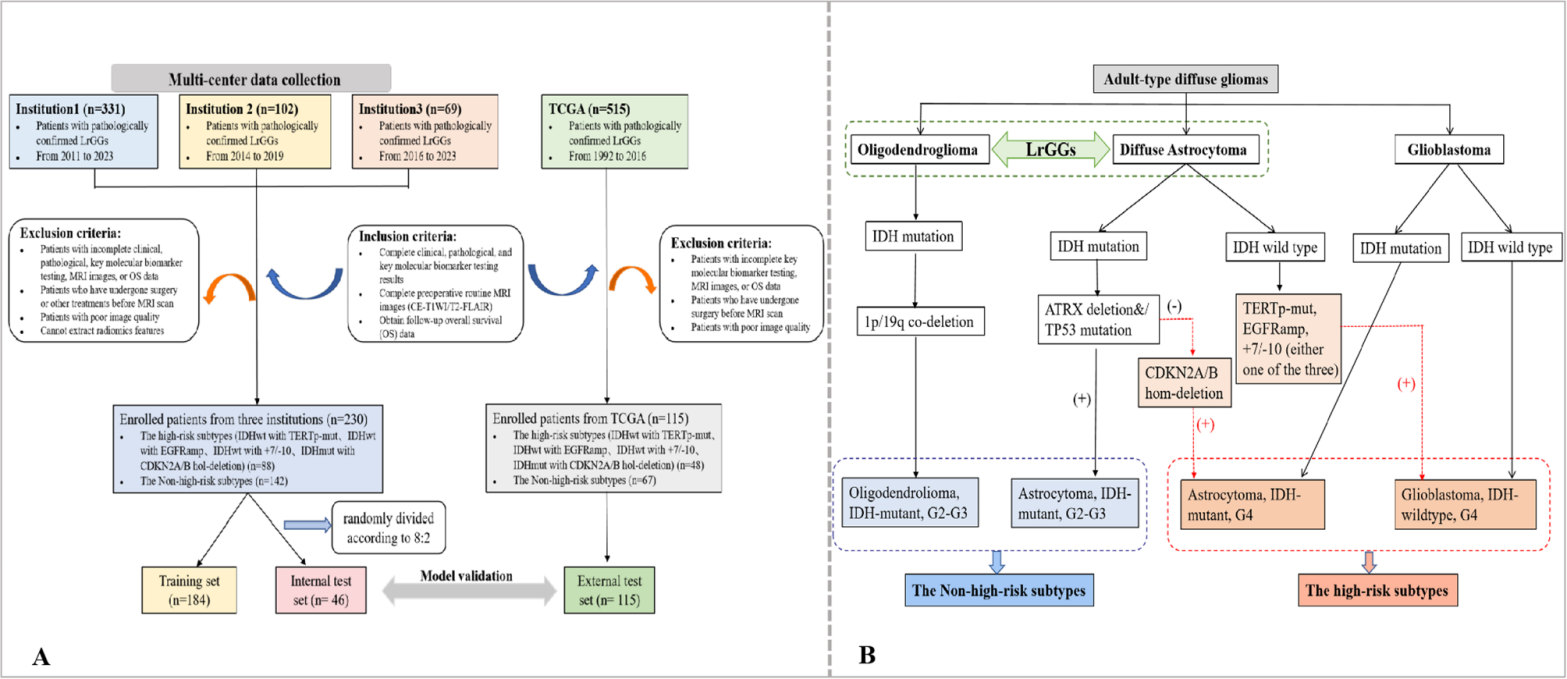
(**A**) Flowchart of patient selection in this study. (**B**) Schematic diagram of screening for all high-risk molecular subtypes of diffuse gliomas in this study drawn according to the WHO guidelines (2021 edition). IDH: isocitrate dehydrogenase; 1p/19q: short arm of chromosome 1 and long arm of chromosome 19; TERTp-mut: telomerase reverse transcriptase promoter mutation; EGFRamp: epidermal growth factor receptor amplification; +7/-10: chromosome 7 gain and chromosome 10 loss; CDKN2A/B hom-deletion: Cyclin-dependent kinase inhibitor 2 A/B homozygous deletion; G2/3/4: grade2/3/4

In this study, 502 LrGGs patients were recruited from three hospitals in this province. After excluding 272 patients who did not meet the criteria, 230 patients were included in the research cohort. Simultaneously, 515 LrGG patients were selected from the TCGA public database. After excluding 400 of them, 115 patients were finally included in the study. The inclusion criteria included: patients with diffuse glioma whose histological grade was confirmed as grade 2–3 after surgery, possessing complete clinical, pathological, key molecular biomarkers detection results, complete preoperative conventional MRI images including CE-T1WI and T2-FLAIR images, as well as availability of data on OS, OS was delineated as the duration from the surgical pathological diagnosis to either the demise of the patient or the latest follow-up. Exclusion criteria applied to patients with incomplete clinical, pathological, MRI preoperative scan, key or molecular biomarkers detection, or OS data, those who underwent surgery, biopsy, radiotherapy, or chemotherapy prior to MRI examination, as well as those with inadequate image quality such as severe motion artifacts, metal artifacts, and cases with difficult extraction of radiomics features. Ultimately, patients who met the criteria were enrolled and randomly allocated into training and testing sets at an 8:2 ratio for model development (Fig. 1).

### High-risk molecular marker detection

The high-risk molecular subgroups of diffuse gliomas were included according to the criteria outlined in our comprehensive classification process, which was developed in accordance with the 2021 WHO guidelines (Fig. 1). To determine the IDH, CDKN2A/B, and TERTp status of gliomas, the Simlex OUP^®^ FFPE DNA extraction kit (TIB, Shanghai, China) was utilized following standardized DNA extraction procedures. Subsequently, glioma DNA was PCR amplified using the ABI 9700 Life Technology system (Thermo Fisher Scientific, Waltham, MA, USA), and final detection results were obtained through Sanger sequencing performed on the ABI 3500 Life Technology platform (Thermo Fisher Scientific, Waltham, MA, USA). Additionally, the FISH method was employed to determine EGFR amplification status and chromosome 7/10 alterations, and final detection results were obtained using the probe reagent kit (VividFISHTM FISH CEP, GeneCopoire). Furthermore, the BisulFlash™ DNA modification kit (Epigentek, Farmingdale, New York, NY, USA) was used for sodium bisulfite modification to extract DNA samples of brain gliomas, and the DRR006 kit (Takara, Kusatsu, Shiga, Japan) was used for PCR amplification to determine the methylation status of the MGMT promoter.

### Image scanning and segmentation

In this study, head scans were performed using the Signa HDxt, GE Healthcare, USA, 3.0T, and Siemens Skyra 3.0T MRI scanners, utilizing an 8-channel phased array coil. The comprehensive scanning parameters are provided in Supplementary Information 1.

We use CE-T1WI as a template for rigid registration of T2-FLAIR images. We set the range of normalized intensity values for the images to [0-255], adjusting the grayscale values of images acquired from different devices to this same interval., and each layer of MR images should be resampled to achieve a uniform distribution with a pixel spacing of 3 × 3 mm^2^. Manual delineation of the region of interest (ROI) on MRI images was performed using ITK software (http://www.itksnap.org, version 3.6.0). Specifically, the tumor solid area was selected as the ROI on the CE-T1WI image, while carefully excluding the edema area surrounding the tumor. Subsequently, the ROI contour was registered onto the T2-FLAIR image for further analysis. The delineation of the ROI was conducted by a neuroradiologist with over ten years of clinical experience. To guarantee precision and uniformity in outlining, a second neuroradiologist, possessing more than 15 years of expertise, reassessed and verified the delineated ROI, offering additional insights as required. The inter-observer consistency of all delineation results between the two physicians was analyzed by calculating the intra-class correlation coefficient (ICC), and values > 0.75 was considered to indicate good correlation.

### Tumor habitat clustering

This study applied a data-driven K-means clustering method to automatically delineate tumor regions [25–27], dividing the tumor area into several spatially distinct zones characterized by consistent signal intensity patterns across multi-parameter MR images [28–30]. To ensure coherence among T1CE and T2FLAIR within each subregion, we first integrated all sequence image information by compiling the voxels within the segmented masks. Subsequently, We extracted 16 radiomic features for each voxel to capture local information using the Pyradiomics package. The extracted radiomic features are as follows: Firstorder_Entropy, Firstorder_MeanAbsoluteDeviation, Firstorder_Median, Glcm_DifferenceEntropy, Glcm_DifferenceVariance, Glrlm_RunEntropy, Glszm_SizeZoneNonUniformityNormalized, Glcm_DifferenceAverage, Glcm_Imc1, Glcm_Imc2, Glcm_JointEntropy, Glcm_SumEntropy, Glrlm_LongRunEmphasis, Glrlm_RunVariance, Ngtdm_Contrast, and Glcm_InverseVariance. Utilizing the K-means clustering algorithm with squared Euclidean distances based on voxel intensities and voxel-level radiomics features as similarity metrics, we grouped individual voxels within each cluster according to their similarities and differences. Given that determining the optimal number of clusters in a dataset is crucial in K-means clustering, we initially tested cluster numbers ranging from 2 to 10. To ensure consistency across patients, clustering was performed at the cohort level rather than the individual patient level. To ascertain the optimal number of clusters, we evaluated clustering outcomes through 100 iterations using the average Calinski-Harabasz score for each k value. Ultimately, we selected a cluster number of 3, as it exhibited the highest Calinski-Harabasz score, effectively highlighting habitat imaging differences and preventing the development of an overly complex model.

### Feature extraction and feature selection

In terms of feature extraction, we utilized the Pyradiomics package (https://github.com/Radiomics/pyradiomics) for feature extraction. Following the Imaging Biomarker Standardization Initiative (IBSI), 1015 radiomics features were extracted from each region of interest, targeting the habitat region for each sequence. These features can be categorized into the following groups: (1) Shape features group (*n* = 14); (2) First-order statistics features group (*n* = 18); (3) Texture features group, including GLCM (*n* = 22), GLRLM (*n* = 16), GLSZM (*n* = 6), GLDM (*n* = 14), NGTDM (*n* = 5); (4) Filtering features group (*n* = 910).

The specific method of feature selection is as follows: Firstly, Mann-Whitney U test and feature selection are applied to all radiomics features, retaining only features with p-values less than 0.05. For highly correlated features, Spearman rank correlation coefficient is calculated to assess the correlation between features. If two features exhibit a correlation coefficient exceeding 0.9, only one feature is preserved while the other is discarded. To maximize the descriptive capability of features, a greedy recursive elimination strategy is employed for feature filtering, removing features at each iteration with maximum redundancy. Following that, the Least Absolute Shrinkage and Selection Operator (LASSO) regression algorithm is employed to identify the optimal subset of radiomics features. This subset comprises radiomics features with non-zero coefficients corresponding to the best-tuned parameterλ, determined through 10-fold cross-validation.

### Development of high-risk molecular subtype habitat prediction model

Following LASSO feature selection, seven machine learning classifiers including Random Forest (RF), Logistic Regression (LR), Extremely Randomized Trees (Extra Trees), SVM, MLP, XGBoost, and LightGBM were employed for model construction. Three predictive models were built based on these classifiers in the training, internal test, and external test sets: the habitat model, radiomics model, and combined habitat-radiomics model. Performance analysis and comparison were conducted among these three models to select the optimal predictive model. Additionally, after the output of the optimal classifier, the final radiomics score (Radscore) was obtained. Model predictive performance was validated using receiver operating characteristic (ROC) curves, 95% confidence intervals (95% CI), specificity, sensitivity, PPV, NPV, Precision, Recall, F1 and Threshold.

### Construction of habitat prognostic prediction model

**Fig. 2:**
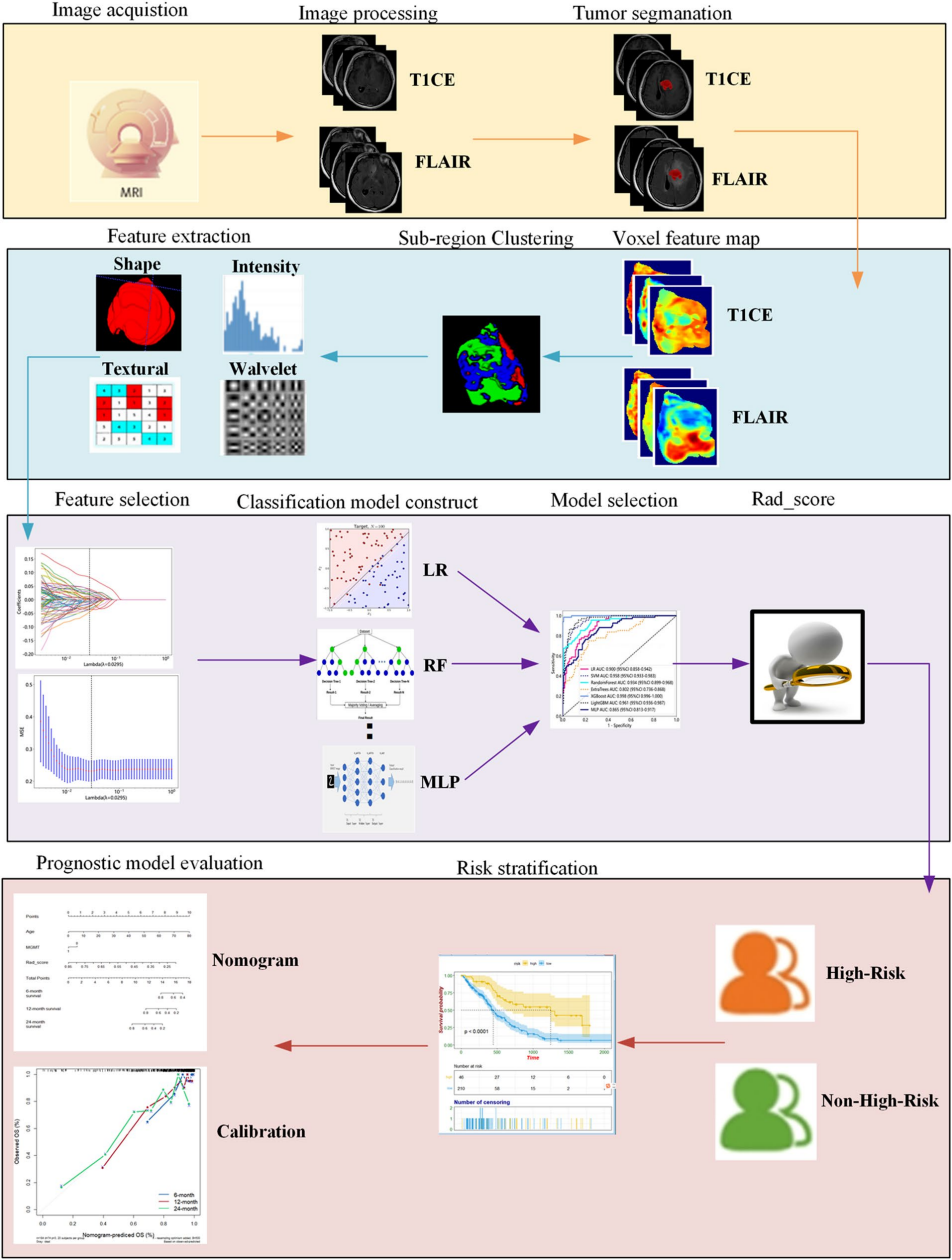
Construction process diagram of habitat model for predicting high-risk molecular subtypes and habitat prognostic model in this study

The habitat prognostic prediction model is constructed by utilizing the radiomic score (Radscore) obtained through Extra Trees classifier output and cross-validation as the risk score. Subsequently, the study sets are uniformly divided into high and low-risk groups based on the median values of Radscore. To assess the efficacy of Radscore as a prognostic radiomics marker, Kaplan-Meier (KM) survival analysis and the Log-rank test are performed to contrast survival disparities between patient groups categorized as high-risk and low-risk. Then, univariate Cox regression and multivariate regression analyses are performed on clinical, pathological, and genetic information to identify risk factor variables significantly associated with overall survival (OS). Radscore is combined with the risk factor variables, and a multi-factor Cox regression is employed to construct a clinical-habitat joint prediction model and nomogram. Concurrently, a clinical prediction model is developed, and the discriminative capacity of the prognostic prediction model is assessed using the concordance index (C-index), followed by a comparison of model performance. Calibration curves are employed to evaluate the alignment between the predicted survival probability from the nomogram and the actual survival probability [31–33]. The process of constructing the habitat prediction model and the prognostic prediction model for the high-risk molecular subtypes of LrGGs in this study is shown in Fig. 2.

### Statistical analysis

R version 4.2.2 (https://www.R-project.org/) was used for statistical analysis. The “Survival” package was employed for univariate and multivariate Cox regression analysis and KM survival analysis. The “rms” package was used to plot column charts. Performance of the predictive model for high-risk molecular subtypes was assessed using the area under the curve (AUC), 95% confidence intervals (CI), specificity, sensitivity, positive predictive value (PPV), negative predictive value (NPV), precision, recall, F1 score and threshold, while the prognostic model’s performance was evaluated using the C-index. CI were calculated using 1000 bootstrap samples, and calibration curves and Hosmer-Lemeshow tests were utilized to assess the actual probability of the model. All statistical analyses were conducted using a two-sided approach, with a significance level set at *P* < 0.05.

## Results

### Patient characteristics analysis

This study collected relevant clinical features of patients with LrGGs from three hospitals and TCGA database, including demographic characteristics, pathological features, and molecular features. Table 1 shows some clinical features of high-risk molecular subtypes and non-high-risk molecular subtypes in the training, internal test and external test sets. Among them, in the training set, age, histological grade, and treatment methods showed statistical differences in the distribution between the two subtypes. In the external test set, age, histological grade, O⁶-methylguanine-DNA methyltransferase (MGMT) methylation status, and treatment methods all had statistically significant differences in the distribution between the two subtypes. However, in the internal test set, none of the above factors had a statistical difference.

**Table 1:** Clinical characteristics of patients in three cohorts

Characteristi-cs	Training set(*n* = 184)		Test statistics	*P* values	Internal test set(*n* = 46)		Test statistics	*P* values	External test set(*n* = 115)		Test statistics	*P* values
	High-risk subtype(*n* = 68)	Non-high-risk subtype(*n* = 116)			High-risk subtype(*n* = 20)	Non-high-risk subtype(*n* = 26)			High-risk subtype(*n* = 48)	Non-high-risk subtype(*n* = 67)		
Age (Years)	49.74 ± 13.36	44.34 ± 17.03	t = 2.24	0.03*	51.35 ± 17.06	46.50 ± 10.62	t = -1.18	0.24	51.23 ± 12.67	51.23 ± 12.67	t = -3.26	0.00*
Gender												
Male	44(64.71)	72(62.07)	χ^2^ = 0.04	0.84	13(65.00)	15(57.69)	χ^2^ = 0.04	0.84	24(50.00)	31(46.27)	χ^2^= 0.16	0.69
Female	24(35.29)	44(37.93)			7(35.00)	11(42.31)			24(50.00)	36(53.73)		
Histological grade												
2	25(36.76)	66(56.90)	χ^2^ = 6.17	0.01*	9(45.00)	15(57.69)	χ^2^ = 0.31	0.58	11(22.92)	42(62.69)	χ^2^= 17.80	0.00*
3	43(63.24)	50(43.10)			11(55.00)	11(42.31)			37(77.08)	25(37.31)		
MGMT-meth status												
Yes	20(29.41)	45(38.79)	χ^2^ = 1.27	0.26	9(45.00)	10(38.46)	χ^2^ = 0.02	0.89	37(77.08)	61(91.04)	χ^2^= 4.33	0.04*
No	48(70.59)	71(61.21)			11(55.00)	16(61.54)			11(22.92)	6(8.96)		
Treatment method												
0	28(41.18)	61(52.58)		0.01*	9(45.00)	10(38.46)		0.77	2(4.17)	18(26.87)		0.00*
1	27(39.71)	41(35.35)			9(45.00)	9(34.62)			31(64.58)	26(38.81)		
2	3(4.41)	9(7.76)			1(5.00)	4(15.38)			4(8.33)	13(19.40)		
3	2(2.94)	4(3.45)			0(0.00)	1(3.85)			7(14.59)	9(13.43)		
4	8(11.76)	1(0.86)			1(5.00)	2(7.69)			4(8.33)	1(1.49)		

*Note P* value < 0.05 and “*” was considered to have a statistical difference. MGMT-meth status: MGMT methylation status, 0: Surgical resection, 1: Surgery with concurrent chemoradiotherapy, 2: Surgery with radiotherapy alone, 3: Surgery with chemotherapy alone, 4: Missing

### Habitat clustering and feature selection

We used the tumor parenchymal region of gliomas as ROI. The habitat technology segmented the regions of interest with different pathophysiological characteristics into several homogeneous or similar sub-regions, namely tumor sub-habitats. Clustering was performed using the K-means algorithm based on voxel-level radiomic features. According to the Calinski-Harabasz score, the optimal number of clusters for the tumor region of interest was determined to be 3, and these values were averaged over 100 repetitions, meaning the tumor ROI was clustered into three sub-habitats. Then, a series of radiomic features, including shape, size, and intensity distribution, were extracted for each sub-habitat.

**Fig. 3:**
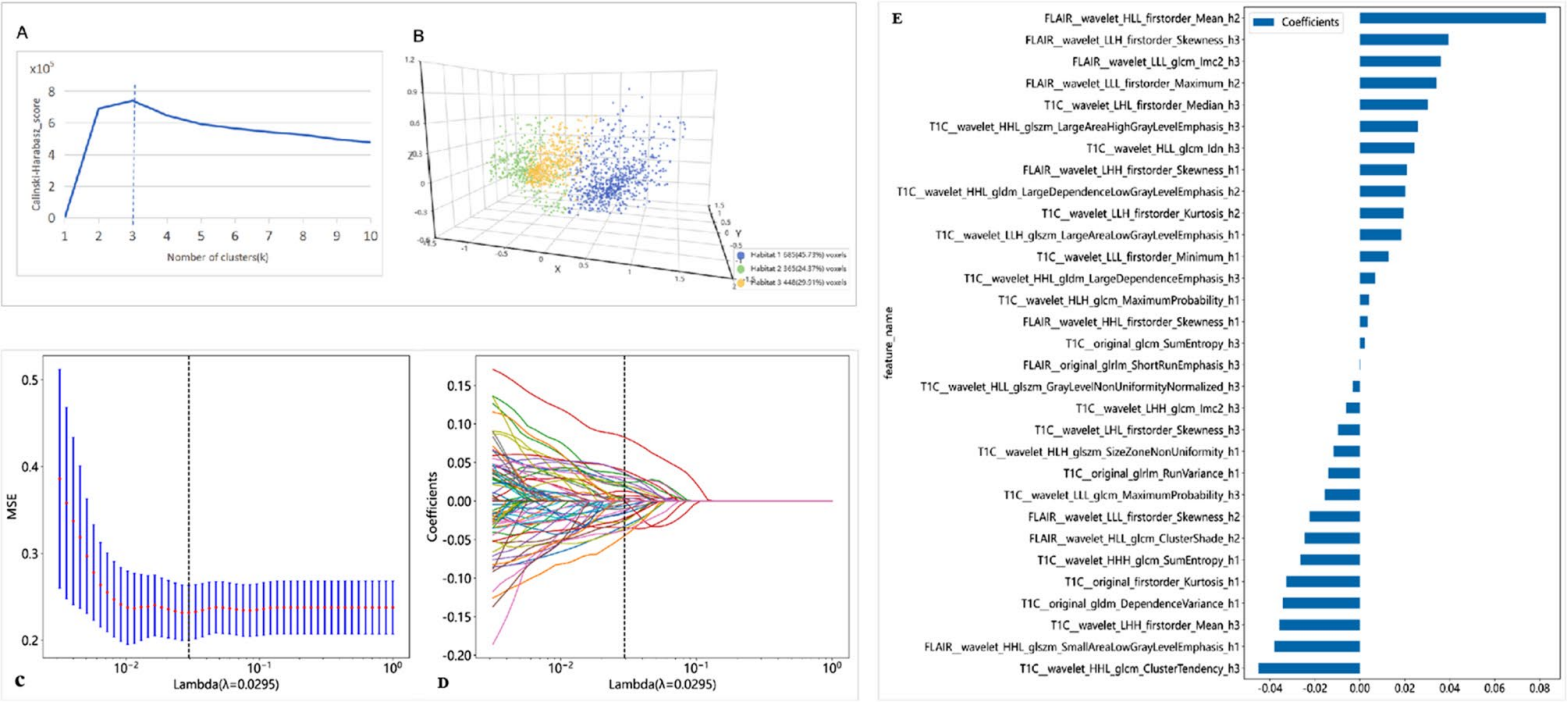
Calinski Harabasz scoring plot (**A**) to determine the optimal number of subareas (habitats). These values are averaged over 100 replicates. (**B**) Habitat clustering, demonstrating the three habitats defined by the normalized. (**C-D**) Selection of Tuning Parameters (Lambda) for the Least Absolute Shrinkage and Selection Operator (LASSO) Regression Model. (**E**) Thirty-one Radiomic Features and their Weights Selected by the LASSO Regression Algorithm

We utilized the Least Absolute Shrinkage and Selection Operator (LASSO) regression algorithm to perform radiomic feature selection and optimize the parameter (λ) screening. Subsequently, 31 optimal features were selected from numerous radiomic features for model construction, among which 10 features originated from T2 - FLAIR images and 21 features from CE - T1WI images. Notably, the features FLAIR_wavelet_HLL_firstorder_Mean_h2 and T1C_wavelet_HHL_glcm_ClusterTendency_h3 made the most significant contributions to model construction (Fig. 3).

### Evaluation of the habitat prediction model for high-risk molecular subtypes

Based on the combination of CE-T1WI and T2-FLAIR scan sequences, three predictive models were constructed using seven machine learning classifiers: the habitat model, the radiomics model, and the combined habitat-radiomics model. Through performance analysis and comparison of these three models, the habitat prediction model based on the Extra Trees classifier ultimately performed best in both the training and testing sets, and was selected as the optimal model for predicting high-risk molecular subtypes of LrGGs.

**Fig. 4:**
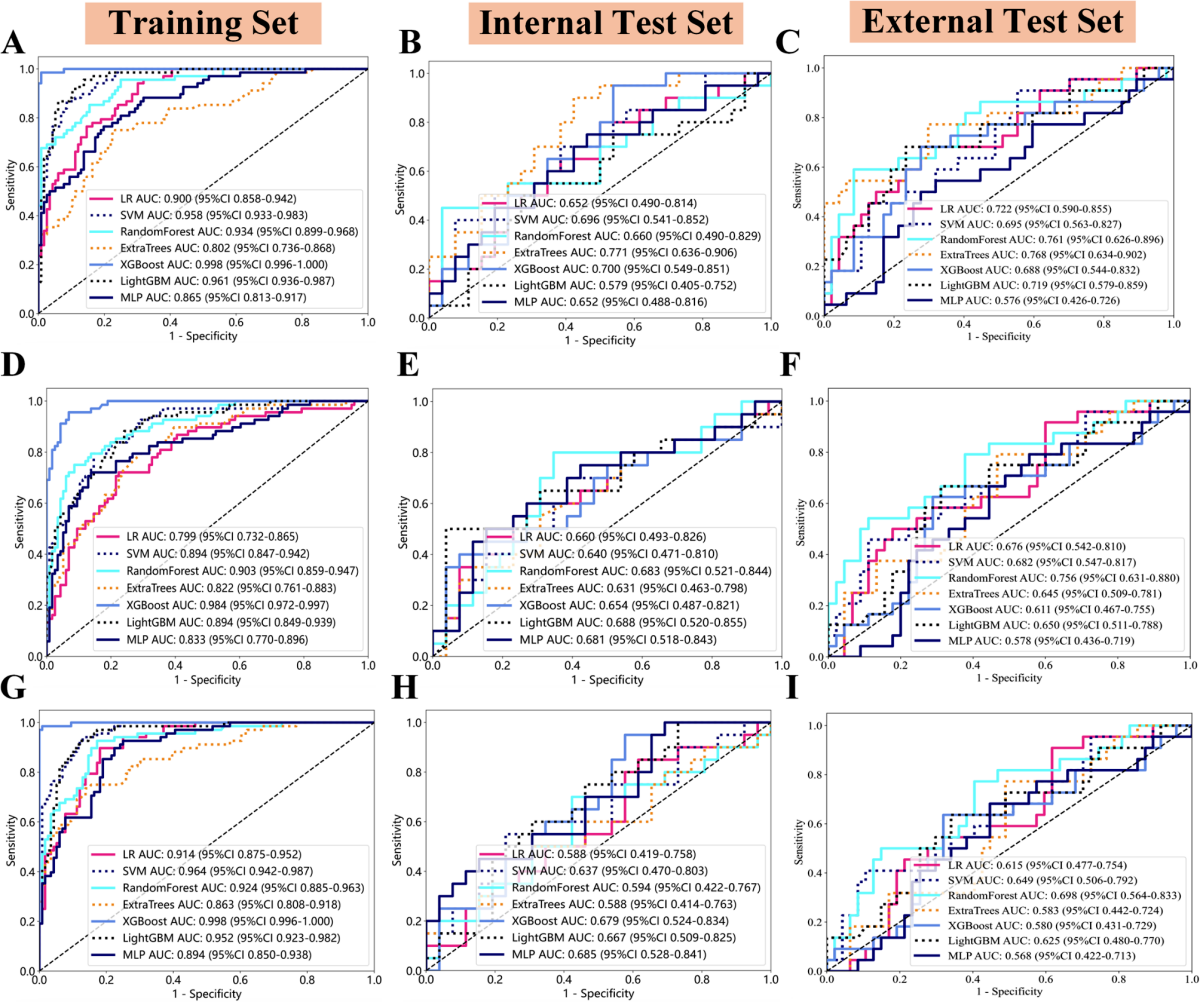
(**A**), (**D**), and (**G**) represent the ROC curves of seven classifier models trained on datasets constructed based on habitat, radiomics, and combined habitat-radiomics, respectively. (**B**), (**E**), and (**H**) represent the corresponding ROC curves of the internal test set, and (**C**), (**F**) and (**I**) represent the corresponding ROC curves of the external test set. Among them, the Extra Trees classifier constructed based on Habitat demonstrates good predictive performance on the study sets

Different predictive models have been meticulously evaluated and compared. The Extra Trees-based habitat model, LightGBM-based radiomics model, and XGBoost-based combined model exhibited the best overall performance in their respective groups. The AUC values and 95% CIs in the training, internal test, and external test sets are as follows: initially, AUCs of 0.802, 0.771, and 0.768 with 95% CIs of 0.996–1.000, 0.636–0.906, and 0.634–0.902; secondly, AUCs of 0.894, 0.688, and 0.650 and 95% CIs of 0.972–0.997, 0.487–0.821, and 0.467–0.755; finally, AUCs of 0.998, 0.679, and 0.580 and 95% CIs of 0.996–1.000, 0.524–0.834, and 0.431–0.729. The Extra Trees-based habitat model had the best performance (Fig. 4). Supplementary Information 2 contains the detailed performance parameters (sensitivity, specificity, PPV, NPV, precision, recall, F1 score, and threshold) of different models.

**Fig. 5:**
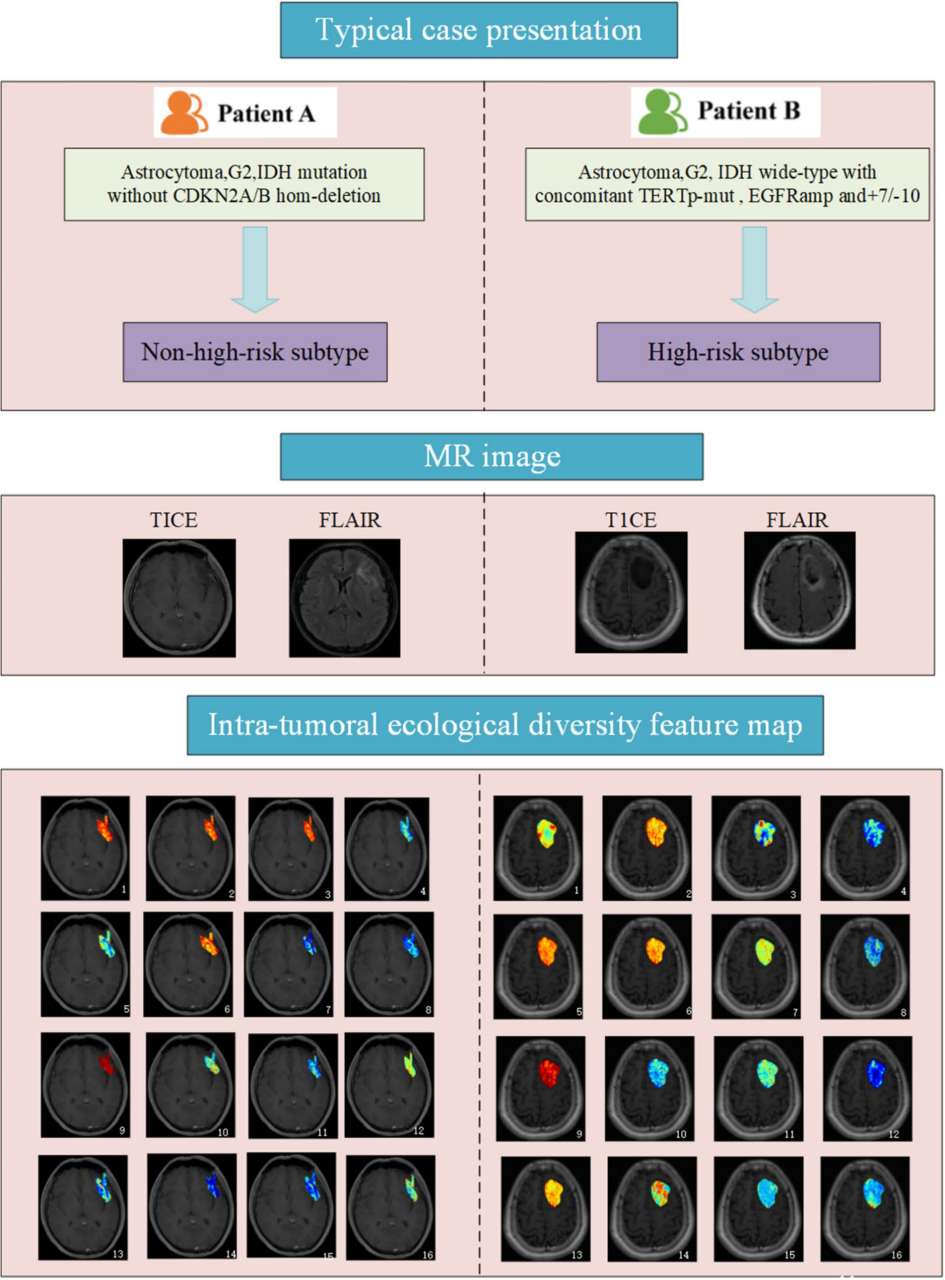
This figure depicts the MRI images and habitat feature maps of two patients with diffuse astrocytoma (WHO grade 2) from different molecular subtypes. Preoperative conventional MRI of patients (**A**) and (**B**) shows similar radiological features, with no enhancement on CE-T1WI images and slightly heterogeneous high signal on T2-FLAIR images. However, the habitat feature maps of these two patients exhibit significant differences

Figure 5 displays the conventional magnetic resonance imaging (MRI) images and habitat radiomics feature distribution maps of two patients with LGGs (histological grade 2) belonging to high-risk and non-high-risk molecular subtypes respectively. In both conventional images, there is no obvious enhancement in T1CE, the tumor body shows a slightly low signal in FLAIR, and the peritumoral edema presents a high signal.However, there are significant differences in the distribution of habitat feature images on T1CE images between the two (judged by the differences in color distribution).

### Evaluation of the prognostic value of the habitat prediction model

We employed both univariate Cox regression and multivariate Cox regression analyses to screen clinical feature variables and subsequently construct a clinical model. Then, we selected habitat radiomics score (Radscore), age, and MGMT methylation status to build a combined clinical-radiomics prediction model (Tabel 2). The results showed that the combined prediction model performed the best (training set: C-index = 0.781, internal testing set: C-index = 0.778, external testing set: C-index = 0.743), followed by the habitat prediction model (training set: C-index = 0.749, internal testing set: C-index = 0.716, external testing set: C-index = 0.707), with the clinical prediction model performing the lowest (training set: C-index = 0.717, internal testing set: C-index = 0.687, external testing set: C-index = 0.649).

**Table 2:** Cox regression analysis of clinical information

Univariate cox	HR	95%CI	*P* value	Multivariable cox	HR	95%CI	*P* value
Age	1.0565	1.037–1.077	< 0.001*	Age	1.0547	1.0358–1.0739	< 0.001*
MGMT	0.5468	0.3389–0.8821	0.013*	MGMT	0.5132	0.3168–0.8312	0.007*
Sex	0.7503	0.4627–1.217	0.244				
Grade	1.5565	0.9775–2.478	0.062				
Treatment Modality	0.8428	0.6544– 1.085	0.185				

*P* value < 0.05 was considered as a significant difference. “*” was considered as a significant difference

**Fig. 6:**
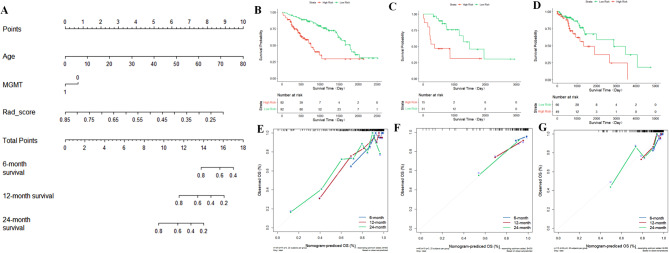
Performance evaluation of the combined prognostic model. (**A**) Nomogram constructed using a combination of Radscore, patient age, and MGMT methylation status to predict 1-year, 2-year, and 3-year overall survival in patients with LrGGs. (**B**–**G**) Represents the calibration curves of combined prognostic model for 1-year, 2-year, and 3-year survival periods in the training, internal test and external test sets, respectively. The gray diagonal line represents the ideal standard, with closer proximity indicating better evaluation performance. Patients with LrGGs are divided into high and low-risk groups based on Radscore thresholds (green curve represents the low-risk group, red represents the high-risk group). Results show that the p-values for all the study sets are < 0.05, indicating statistically significant differences

In addition, we constructed a nomogram of the combined model based on patients’ age, MGMT methylation status, and Radscore to predict the 1 - year, 2 - year, and 3 - year survival probabilities of patients with LrGGs. The calibration curves demonstrated good calibration performance in the training set, internal test set, and external test set. Moreover, there are significant differences in survival probabilities between the high - risk and low - risk groups divided according to the habitat Radscore threshold, with *P* < 0.05 (Fig. 6). The Radscore generated by the habitat prediction model can serve as an independent prognostic indicator for predicting the prognosis of patients with LGGs.

## Discussion

Lower-grade gliomas (LrGGs, WHO grades 2–3) are a complex heterogeneous group of tumors, exhibiting varying clinical courses and prognostic outcomes. Traditional histopathological grading often fails to accurately reflect the biological behavior differences, which are more determined by the molecular subtypes of the tumor [34–36]. Among LrGGs, there exist a minority of high-risk molecular subtypes with malignant transformation potential, associated with adverse clinical outcomes and shorter survival prognosis [37]. This study aims to develop a habitat model utilizing preoperative MRI images to non-invasively predict high-risk molecular subtypes in LrGGs. Furthermore, an accompanying habitat-based prognostic prediction model was established to facilitate early risk assessment of LrGGs. This study is of significant importance for early non-invasive warning of malignant transformation in LrGGs, survival prognosis assessment, and preoperative risk stratification management.

Currently, research on intelligent imaging predicting molecular subtypes of brain gliomas mostly focuses on individual molecular subtypes [38–42], with few studies considering the four high-risk molecular subtypes in LrGGs as a whole (i.e., IDH-wildtype with TERTp-mutation, IDH-wildtype with EGFR amplification, IDH-wildtype with + 7/-10 alterations, IDH-mutation with CDKN2A/B homozygous deletion). For instance, Zhang et al. [43] developed a deep learning model using conventional MRI images from the TCGA/TCIA database to predict the CDKN2A/B co-deletion status in IDH-mutant astrocytomas. Wu et al. [22] developed a habitat analysis model based on preoperative MRI perfusion imaging, enabling prediction of IDH mutation status and survival prognosis in high-grade gliomas. In contrast, our study examines the four high-risk molecular subtypes of LrGGs as a whole and comprehensively investigates the key molecular markers associated with these four high-risk subtypes. To our knowledge, research utilizing habitat technology to predict the four high-risk molecular subtypes of LrGGs is extremely limited, which is also one of the innovative aspects of this study.

One of the highlights of this study lies in integrating habitat technology with high-risk molecular subtyping and survival assessment for analysis and exploration. Not only does this study achieve non-invasive prediction of the four high-risk molecular subtypes using habitat technology, but it also successfully assesses the overall survival of patients with different molecular subtypes, yielding excellent results. This study accomplished a comprehensive evaluation of brain gliomas using habitat MRI imaging technology, offering clinicians additional valuable reference information, thereby providing incremental value to glioma treatment decisions.

This study employed seven categories for the construction of the prediction model: LR, SVM, RandomForest, Extra Trees, XGBoost, LightGBM, and MLP, among which the habitat model based on the Extra Trees classifier achieved the best predictive performance in predicting high-risk molecular subtypes of LrGGs, with AUC values of 0.802, 0.771 and 0.768 in the training set, internal test set, and external test set, respectively. Compared to other algorithms such as Random Forest, Extra Trees are more efficient. This is because during node splitting, it doesn’t just randomly select features but chooses the optimal split points from random subsets of features, reducing computational costs. Additionally, Extra Trees introduce more randomness, which helps reduce model overfitting to training data, especially in high-dimensional datasets. Moreover, they exhibit strong robustness to noisy data and missing features. Zhang et al. [44]constructed a postoperative recovery prediction model for cervical spondylosis using the Extra Trees classifier, which yielded favorable predictive performance. AUC for the internal validation cohort and the external validation cohort reached 0.81 and 0.75, respectively. Currently, studies on high-risk molecular subtypes of gliomas primarily focus on preoperative prediction of individual molecular subtypes. For instance, Zhang et al. [45]developed a radiomics model based on multi-parameter MRI images to predict the TERT promoter mutation status in glioblastoma patients. The results showed that the radiomics model constructed using logistic regression achieved AUC values of 0.816, 0.812, and 0.823 in the training set, internal test set, and external test set, respectively. Compared to the performance of the habitat model based on the Extra Trees classifier in this study, the model performance in our study is slightly lower. Considering that the prediction target in our study is the overall classification of four high-risk molecular subtypes, as opposed to a single high-risk subtype, comparisons of the performance of the Extra Trees classifier with results reported in other studies are somewhat limited due to differences in data characteristics, experimental setups, and evaluation metrics among different studies. Furthermore, the habitat model achieves high evaluation performance in predicting the overall survival of patients with LrGGs at 1, 2, and 3 years. Based on the habitat radiomics score (Radscore) threshold, LrGGs patients were classified into high and low-risk groups, which showed a significant correlation with overall survival (OS). These findings demonstrate the feasibility and effectiveness of using habitat technology for predicting molecular subtypes and prognosis of LrGGs. They also confirm our hypothesis that the four high-risk molecular subtypes indeed have worse survival outcomes compared to non-high-risk subtypes, with statistical differences. This study validates the comprehensive assessment of gliomas using habitat methods, providing valuable reference information for clinical practice and assisting in precision diagnosis and treatment. It holds profound significance for advancing clinical decision-making.

However, this study has some limitations. First, the collected data has an insufficient sample size. Future work will center on gathering more multicenter samples and conducting prospective studies. Second, the research design for survival prognosis prediction is suboptimal as it neglects patients’ comprehensive clinical details like specific treatment particulars such as surgical resection extent and radiotherapy or chemotherapy regimen specificity. It’s crucial to refine the prognosis prediction model by incorporating more survival-related clinical factors. We’ll strive to collect more detailed clinical information in the later study period. Third, the model has limitations in practical applications, especially when dealing with noisy data. These limitations stem from the structural and decision-making characteristics of the Extra Trees classifier, which is more sensitive to data purity. Future research may require the introduction of data preprocessing and the optimization of the model structure to improve its robustness in complex practical scenarios.

## Conclusion

This study developed a habitat analysis model based on preoperative conventional MRI imaging to simultaneously predict high-risk molecular subtypes and assess survival prognosis of LrGGs. Additionally, it demonstrated that the habitat Radscore could serve as an independent prognostic risk marker for LrGGs patients. This holds significant importance for early non-invasive warning of malignant transformation and early risk assessment in LrGGs, playing a proactive role in timely selection of intervention windows, adjustment of treatment plans, and precision diagnosis and treatment in clinical practice.

## Electronic supplementary material

Below is the link to the electronic supplementary material.

Supplementary Material 1

Supplementary Material 2

## Data Availability

No datasets were generated or analysed during the current study.
