# Supplementary material for: MRI-based habitat imaging predicts high-risk molecular subtypes and early risk assessment of lower-grade gliomas
Source: Cancer Imaging. 2025 Mar 28;25:43. doi: 10.1186/s40644-025-00838-4 (PMC11951782; doi:10.1186/s40644-025-00838-4)
Supplement: Supplementary file 1 — Supplementary Material 1 [file 40644_2025_838_MOESM1_ESM.pdf]

The specific scanning parameters for the GE Healthcare 3.0T MRI scanner were as follows: for CE-T1WI acquisition: repetition time/echo time (TR/TE) of 1950 ms / 47.6 ms, field of view (FOV) of 240 mm, slice thickness/gap of 5.0 mm/1.5 mm, matrix size of 256 × 256; for T2-FLAIR acquisition: TR/TE of 8000 ms/95 ms, FOV of 240 mm, slice thickness/gap of 5.0 mm/1.5 mm, matrix size of 256×256.

The specific scanning parameters for the Siemens Skyra 3.0T MRI scanner were: for CE-T1WI acquisition: TR/TE of 2000ms / 42ms, FOV of 230mm, slice thickness/gap of 5.5mm/ 1.3mm, matrix size of 256 × 256; for T2-FLAIR acquisition: TR/TE of 8000ms/ 88ms, slice thickness/gap of 5.5mm/1.3mm, FOV of 230mm, matrix size of 256 × 256. Gadopentetate dimeglumine injection was used for all T1-weighted contrast-enhanced scans, administered via the cubital vein at a dose of 0.1 mmol/kg, at a rate of 2 to 3 ml/s.
