# Supplementary material for: MRI-based habitat imaging predicts high-risk molecular subtypes and early risk assessment of lower-grade gliomas
Source: Cancer Imaging. 2025 Mar 28;25:43. doi: 10.1186/s40644-025-00838-4 (PMC11951782; doi:10.1186/s40644-025-00838-4)
Supplement: Supplementary file 2 — Supplementary Material 2 [file 40644_2025_838_MOESM2_ESM.pdf]

Tab.1 The performance of the radiomics models for high-risk molecular subtypes prediction

study set	model_name	Accuracy	AUC	95% CI	Sensitivity	Specificity	NPV	Precision	F1
training set	LR	0.750	0.799	0.7319 - 0.8655	0.706	0.776	0.818	0.649	0.676
internal test set	LR	0.652	0.660	0.4932 - 0.8260	0.550	0.731	0.679	0.611	0.579
external test set	LR	0.696	0.676	0.5420 - 0.8098	0.458	0.822	0.740	0.579	0.512
training set	SVM	0.826	0.894	0.8466 - 0.9422	0.779	0.853	0.868	0.757	0.768
internal test set	SVM	0.630	0.640	0.4712 - 0.8096	0.650	0.615	0.696	0.565	0.605
external test set	SVM	0.725	0.682	0.5475 - 0.8173	0.417	0.889	0.741	0.667	0.513
training set	RandomForest	0.848	0.903	0.8593 - 0.9474	0.735	0.914	0.855	0.833	0.781
internal test set	RandomForest	0.696	0.683	0.5214 - 0.8440	0.750	0.654	0.773	0.625	0.682
external test set	RandomForest	0.754	0.756	0.6310 - 0.8801	0.500	0.889	0.769	0.706	0.585
training set	ExtraTrees	0.750	0.822	0.7613 - 0.8832	0.721	0.767	0.824	0.645	0.681
internal test set	ExtraTrees	0.587	0.631	0.4632 - 0.7983	0.750	0.462	0.706	0.517	0.612
external test set	ExtraTrees	0.609	0.645	0.5094 - 0.7813	0.750	0.533	0.800	0.462	0.571
training set	XGBoost	0.935	0.984	0.9716 - 0.9966	0.941	0.931	0.964	0.889	0.914
internal test set	XGBoost	0.674	0.654	0.4866 - 0.8211	0.350	0.923	0.649	0.778	0.483
external test set	XGBoost	0.667	0.611	0.4669 - 0.7553	0.583	0.711	0.762	0.519	0.549
training set	LightGBM	0.799	0.894	0.8485 - 0.9393	0.868	0.759	0.907	0.678	0.761
internal test set	LightGBM	0.739	0.687	0.5201 - 0.8549	0.450	0.962	0.694	0.900	0.600
external test set	LightGBM	0.667	0.650	0.5107 - 0.7883	0.625	0.689	0.775	0.517	0.566
training set	MLP	0.804	0.833	0.7701 - 0.8960	0.706	0.862	0.833	0.750	0.727
internal test set	MLP	0.674	0.681	0.5181 - 0.8434	0.450	0.846	0.667	0.692	0.545
external test set	MLP	0.551	0.578	0.4361 - 0.7195	0.750	0.444	0.769	0.419	0.537

Tab.2 The performance of the combined habitat-radiomics models for high-risk molecular subtypes prediction

study set	model_name	Accuracy	AUC	95% CI	Sensitivity	Specificity	NPV	Precision	F1
training set	LR	0.842	0.914	0.8746 - 0.9524	0.882	0.819	0.922	0.741	0.805
internal test set	LR	0.565	0.588	0.4189 - 0.7580	0.800	0.385	0.714	0.500	0.615
external test set	LR	0.536	0.615	0.4767 - 0.7535	0.864	0.383	0.857	0.396	0.543
training set	SVM	0.891	0.964	0.9416 - 0.9866	0.926	0.871	0.953	0.808	0.863
internal test set	SVM	0.652	0.637	0.4698 - 0.8033	0.500	0.769	0.667	0.625	0.556
external test set	SVM	0.725	0.649	0.5063 - 0.7916	0.364	0.894	0.750	0.615	0.457
training set	RandomForest	0.859	0.924	0.8855 - 0.9630	0.912	0.828	0.941	0.756	0.827
internal test set	RandomForest	0.609	0.594	0.4219 - 0.7665	0.650	0.577	0.682	0.542	0.591
external test set	RandomForest	0.638	0.698	0.5640 - 0.8325	0.727	0.596	0.824	0.457	0.561
training set	ExtraTrees	0.810	0.863	0.8084 - 0.9181	0.721	0.862	0.840	0.754	0.737
internal test set	ExtraTrees	0.630	0.588	0.4135 - 0.7634	0.400	0.808	0.636	0.615	0.485
external test set	ExtraTrees	0.580	0.583	0.4421 - 0.7242	0.727	0.511	0.800	0.410	0.525
training set	XGBoost	0.984	0.998	0.9956 - 1.0000	0.971	0.991	0.983	0.985	0.978
internal test set	XGBoost	0.630	0.679	0.5236 - 0.8341	0.900	0.423	0.846	0.545	0.679
external test set	XGBoost	0.652	0.580	0.4311 - 0.7295	0.591	0.681	0.780	0.464	0.520
training set	LightGBM	0.891	0.952	0.9227 - 0.9816	0.912	0.879	0.944	0.816	0.861
internal test set	LightGBM	0.630	0.667	0.5092 - 0.8254	0.550	0.692	0.667	0.579	0.564
external test set	LightGBM	0.638	0.625	0.4801 - 0.7704	0.591	0.660	0.775	0.448	0.510
training set	MLP	0.810	0.894	0.8501 - 0.9385	0.912	0.750	0.935	0.681	0.780
internal test set	MLP	0.587	0.685	0.5280 - 0.8412	0.950	0.308	0.889	0.514	0.667
external test set	MLP	0.580	0.568	0.4221 - 0.7133	0.636	0.553	0.765	0.400	0.491

Tab.3 The performance of the habitat models for high-risk molecular subtypes prediction

study set	model_name	Accuracy	AUC	95% CI	Sensitivity	Specificity	NPV	Precision	F1
training set	LR	0.783	0.900	0.8583 - 0.9419	0.926	0.698	0.942	0.643	0.759

study set	model_name	Accuracy	AUC	95% CI	Sensitivity	Specificity	NPV	Precision	F1
internal test set	LR	0.609	0.652	0.4899 - 0.8140	0.750	0.500	0.722	0.536	0.625
external test set	LR	0.696	0.722	0.5899 - 0.8550	0.636	0.723	0.810	0.519	0.571
training set	SVM	0.891	0.958	0.9335 - 0.9826	0.853	0.914	0.914	0.853	0.853
internal test set	SVM	0.674	0.696	0.5406 - 0.8517	0.350	0.923	0.649	0.778	0.483
external test set	SVM	0.580	0.695	0.5634 - 0.8273	0.864	0.447	0.875	0.422	0.567
training set	RandomForest	0.821	0.934	0.8988 - 0.9684	0.941	0.750	0.956	0.688	0.795
internal test set	RandomForest	0.717	0.660	0.4901 - 0.8291	0.400	0.962	0.676	0.889	0.552
external test set	RandomForest	0.797	0.761	0.6263 - 0.8959	0.545	0.915	0.811	0.750	0.632
training set	ExtraTrees	0.755	0.802	0.7356 - 0.8680	0.735	0.767	0.832	0.649	0.690
internal test set	ExtraTrees	0.696	0.771	0.6364 - 0.9059	0.850	0.577	0.833	0.607	0.708
external test set	ExtraTrees	0.797	0.768	0.6336 - 0.9022	0.500	0.936	0.800	0.786	0.611
training set	XGBoost	0.984	0.998	0.9959 - 1.0000	0.971	0.991	0.983	0.985	0.978
internal test set	XGBoost	0.652	0.700	0.5488 - 0.8512	0.900	0.462	0.857	0.562	0.692
external test set	XGBoost	0.696	0.688	0.5437 - 0.8315	0.636	0.723	0.810	0.519	0.571
training set	LightGBM	0.891	0.961	0.9357 - 0.9868	0.941	0.862	0.962	0.800	0.865
internal test set	LightGBM	0.609	0.579	0.4052 - 0.7525	0.500	0.692	0.643	0.556	0.526
external test set	LightGBM	0.725	0.719	0.5793 - 0.8588	0.636	0.766	0.818	0.560	0.596
training set	MLP	0.783	0.865	0.8125 - 0.9175	0.750	0.802	0.845	0.689	0.718
internal test set	MLP	0.609	0.652	0.4882 - 0.8156	0.700	0.538	0.700	0.538	0.609
external test set	MLP	0.623	0.576	0.4264 - 0.7264	0.500	0.681	0.744	0.423	0.458
